# Intestinal Ultrasound: Advancing Towards Broader Adoption—Insights from a National Survey in Turkey

**DOI:** 10.3390/jcm14144817

**Published:** 2025-07-08

**Authors:** Gülden Bilican, Tarkan Karakan, Ödül Eğritaş Gürkan, Mehmet Cindoruk, Charlotte Hedin, Haider Sabhan, Ayşe Can, Stephan L. Haas

**Affiliations:** 1 Department of Gastroenterology, Faculty of Medicine, Gazi University, Ankara 06560, Turkey; tkarakan@gmail.com (T.K.); mcindoruk@gazi.edu.tr (M.C.); 2 Department of Pediatric Gastroenterology, Faculty of Medicine, Gazi University, Ankara 06560, Turkey; odulmd2003@yahoo.com (Ö.E.G.); aysegunescan@hotmail.com (A.C.); 3 Department of Medicine, Solna, Department of Gastroenterology, Dermatovenereology and Rheumatology, Karolinska Institute, Centre for Digestive Health, Karolinska University Hospital, 17164 Stockholm, Sweden; charlotte.hedin@ki.se (C.H.); haider.sabhan@capiostgoran.se (H.S.); stephan.haas@ki.se (S.L.H.)

**Keywords:** intestinal ultrasound, inflammatory bowel disease, survey

## Abstract

**Objective**: Intestinal ultrasound (IUS) is increasingly valued as a noninvasive tool for inflammatory bowel disease (IBD) management, offering real-time, radiation-free assessment of bowel wall thickness, vascularity, and complications. While IUS is widely adopted in Europe, data on its use in Turkey is scarce. This study aims to address this gap. **Methods**: A nationwide, cross-sectional survey was conducted targeting 817 adult and 150 pediatric gastroenterologists in Turkey. The survey included 26 structured questions on demographics, familiarity with and use of IUS, and barriers to implementation. **Results**: A total of 191 gastroenterologists participated in this survey, with 56% being adult gastroenterologists (n = 107) and 44% pediatric gastroenterologists (n = 84). Regarding whether they participated in IUS training, 73% (n = 140) of the 191 respondents stated they had not received training. There were notable differences in how IUS was utilized among gastroenterologists: 29% (n = 31) of adult gastroenterologists performed IUS independently, compared to just 2% (n = 2) of pediatric gastroenterologists (*p* < 0.001). In total, 63% (n = 67) of adult gastroenterologists and 46% (n = 39) of pediatric gastroenterologists reported not using IUS. Altogether, 94% (n = 179) emphasized the necessity of educational opportunities, and 86% (n = 165) favored national guidelines. **Conclusions**: Our findings reveal that the current application of IUS in Turkey fails to correspond with its expected advantages in managing IBD. Limited educational opportunities are a major challenge, emphasizing the necessity for coordinated educational programs and national guidelines. The expanded adoption of the IUS might significantly improve Turkey’s management of IBD. **What is known**: Intestinal ultrasound (IUS) is a non-invasive, cost-effective, and reliable imaging method increasingly recognized for its utility in diagnosing and monitoring inflammatory bowel disease (IBD). **What is new**: This is the first national survey assessing the awareness, usage patterns, and barriers to the adoption of IUS among gastroenterologists in Turkey. The study highlights significant gaps in training opportunities while also identifying strategies to promote IUS integration into routine clinical practice. The findings may encourage similar efforts in other regions where IUS remains underutilized, ultimately improving IBD management and patient outcomes globally.

## 1. Introduction

Inflammatory bowel disease (IBD), which includes Crohn’s disease (CD) and ulcerative colitis (UC), is a chronic relapsing disorder that significantly affects patients’ quality of life and requires lifelong management. Traditionally, assessment of disease activity has relied heavily on clinical symptoms and invasive procedures such as endoscopy and cross-sectional imaging, including magnetic resonance enterography (MRE) and computed tomography enterography (CTE). While these methods provide comprehensive information about the extent and severity of inflammation, they have limitations, including procedural risks, the need for bowel preparation, and high costs [1].

Intestinal ultrasound (IUS) is increasingly recognized as a valuable, noninvasive imaging tool for IBD management. It allows for real-time, radiation-free assessment of bowel wall thickness, vascularity, and complications, such as strictures and abscesses, without the need for sedation or bowel preparation [2,3]. Multiple studies have demonstrated IUS reaches accuracy levels similar to traditional imaging modalities [4]. For instance, IUS has shown comparable sensitivity and specificity to MRE for assessing ileal disease and superior performance in evaluating colonic inflammation [5]. Additionally, IUS’s portability and cost-effectiveness make it a practical option for frequent monitoring, particularly in the context of a treat-to-target strategy that aims for deep remission, including both clinical and endoscopic healing. Furthermore, IUS has the advantage of being well-tolerated by patients and lacks the risks associated with radiation, making it suitable for all patients, especially in settings where minimizing radiation exposure is crucial, such as in pediatric and pregnant populations [6]. ECCO guidelines recommend the application of IUS for monitoring disease activity and assessing response to treatment [7].

IUS is a diagnostic method that requires adherence to numerous standards and necessitates specialized training [8]. Despite the advantages and growing adoption of IUS in Europe and other regions, its use remains limited in Turkey. Until now, data regarding the application of IUS in Turkey has been scarce, which this study aims to address.

Our objective is to assess the current awareness, knowledge, and use of IUS among adult and pediatric gastroenterologists in Turkey. By identifying barriers and training needs, we seek to propose strategies that can facilitate the broader integration of IUS into routine clinical practice. This research could have important implications for improving IBD management, making disease monitoring more accessible and efficient, and ultimately, improving patient outcomes.

## 2. Methods

### 2.1. Study Design and Participants

We conducted a cross-sectional survey of adult and pediatric gastroenterologists in Turkey. The survey was disseminated via email with the assistance of the Turkish Society of Gastroenterology and remained open to responses from 1 November to 14 November, 2024. A total of 817 adults and 150 pediatric gastroenterologists were contacted via email, with a reminder email sent seven days later (Supplementary Materials Figure S1). A total of 191 gastroenterologists ultimately completed the survey. The survey was distributed through Google Forms (Google LLC, Mountain View, CA, USA), providing an accessible platform for respondents.

### 2.2. Survey Instrument

The survey consisted of 26 structured questions divided into three sections: (1) demographic information, (2) familiarity with and usage of IUS, and (3) experience with hepatobiliary ultrasound (HBUS) (Supplementary Materials Table S1). In this survey, the term IUS refers specifically to conventional transabdominal IUS. The questions covered the frequency of IUS use, perceived barriers, prior training, and imaging preferences in the management of IBD. The survey was developed based on a literature review and refined through expert consultation to ensure clarity and relevance.

### 2.3. Statistical Method

Descriptive statistics were used to present the data, with categorical variables shown as numbers (n) and percentages (%). The comparison of categorical variables between groups was performed using the Pearson’s chi-square test or Fisher’s exact test, as appropriate. When expected cell counts in contingency tables were below five, a two-sided Fisher’s exact test was applied instead of the Pearson’s chi-square test.

A Type 1 error rate (alpha) of 0.05 was accepted. Data analysis was conducted using SPSS Statistics, version 29.0 (IBM Corp., Chicago, IL, USA).

## 3. Results

### 3.1. Demographics

A total of 191 gastroenterologists participated in this survey, with 56% being adult gastroenterologists (n = 107) and 44% pediatric gastroenterologists (n = 84). Demographic and professional characteristics of the respondents, including age, gender, years of practice, academic titles, areas of special interest, and institutional affiliations, are summarized in Table 1.

### 3.2. Intestinal Ultrasound Usage

**Figure 1:**
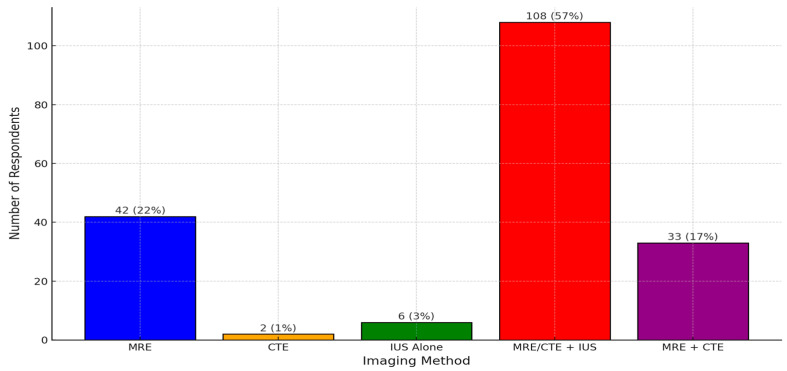
Imaging methods used for IBD diagnosis.

In addition to colonoscopy, various imaging methods were used for IBD diagnosis, with the combination of IUS with either MRE or CTE being the most commonly reported method (Figure 1).

For IBD monitoring, 95% (n = 181) of respondents used colonoscopy, with 78% (n = 148) relying on fecal calprotectin as a biochemical marker. MRE was used by 61% (n = 116), while 30% (n = 58) employed IUS. Additionally, 89% (n = 169) used clinical scoring systems, such as the ulcerative colitis activity index and the Crohn’s disease activity index, to assess disease activity.

When asked about the use of IUS, responses were categorized as follows: 27% (n = 52) of respondents requested it as a radiology consultation, 17% (n = 33) performed it themselves, and 56% (n = 106) reported not using IUS. Among those who used IUS, 9% (n = 18) used it rarely, 21% (n = 40) used it frequently, and 14% (n = 27) used it for every patient.

**Figure 2:**
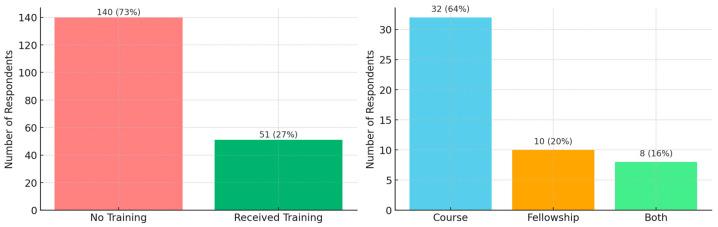
IUS training and training formats.

Figure 2 summarizes the proportion of respondents who received IUS training, along with the formats through which the training was received.

The primary purposes for using IUS were reported as follows: diagnosis in 28% (n = 54), assessment of treatment response in 36% (n = 68), and identification of complications in 35% (n = 66).

**Figure 3:**
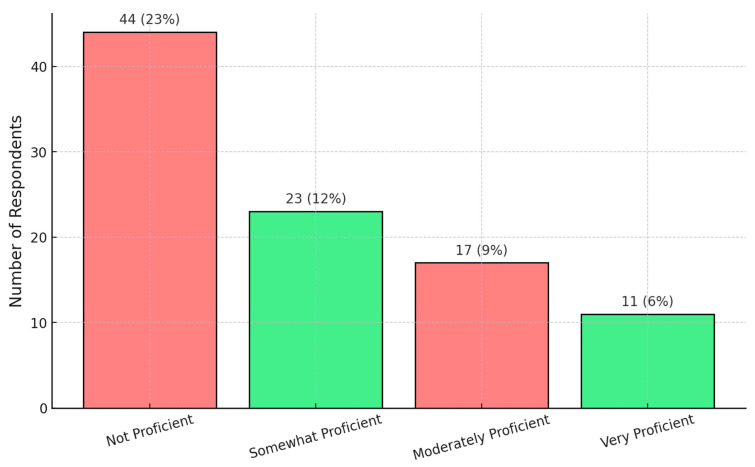
Self-assessed proficiency in using IUS.

Self-assessed proficiency in using IUS showed notable variation among respondents, with the largest proportion reporting low proficiency levels. Further details are presented in Figure 3.

Participants identified several perceived advantages of IUS, with non-invasiveness being the most cited at 95% (n = 182). Other advantages included bedside and outpatient applicability 86% (n = 165), lack of radiation exposure 82% (n = 156), cost-effectiveness 82% (n = 156), and the ability to visualize bowel wall layers and vascularity 77% (n = 147).

**Figure 4:**
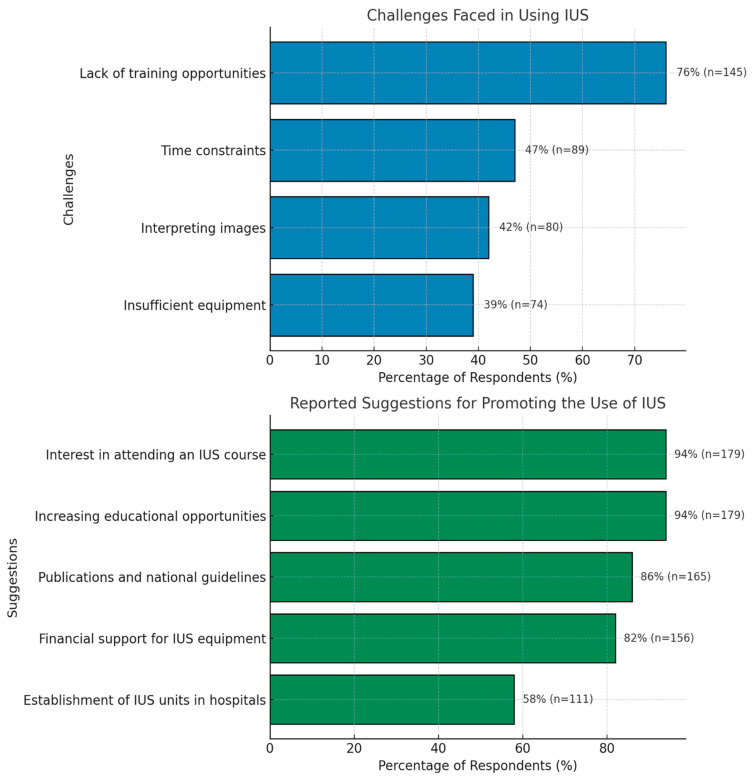
Challenges faced and suggestions for promoting the use of IUS.

Respondents identified several challenges in using IUS, with lack of training opportunities being the most frequently reported issue, followed by time constraints, difficulty interpreting images, and insufficient equipment. Details are shown in Figure 4.

The analysis evaluated the relationship between IUS usage frequency and self-assessed proficiency. Among respondents who reported using IUS rarely, 22% (n = 2) identified themselves as not proficient, while 78% (n = 7) considered themselves proficient. For those who used IUS frequently, 32% (n = 7) were not proficient, and 68% (n = 15) were proficient. Similarly, among those who used IUS for every patient, 24% (n = 4) were not proficient, while 76% (n = 13) were proficient.

This data demonstrates that a higher percentage of those who use IUS more frequently (frequently or for every patient) self-assess as proficient compared to those who use it rarely. However, the association between IUS usage frequency and self-assessed proficiency was not statistically significant (*p* = 0.837).

Participants emphasized several strategies to promote the widespread use of IUS in Turkey, with increasing educational opportunities and attending IUS courses being the most frequently suggested. Other key recommendations included developing publications and national guidelines, providing financial support for equipment, organizing more courses at national congresses, and establishing specialized IUS units in hospitals. Notably, the majority of respondents expressed a strong interest in attending an IUS course if organized in Turkey (Figure 4).

### 3.3. Hepatobiliary Ultrasound Usage

Regarding the use of hepatobiliary ultrasound (HBUS), responses were as follows: 36% (n = 68) reported performing it themselves, 44% (n = 84) requested radiology consultation, and 20% (n = 39) did not use HBUS. Among those who used it, 11% (n = 20) used it rarely, 50% (n = 95) used it frequently, and 18% (n = 35) used it for every patient.

**Figure 5:**
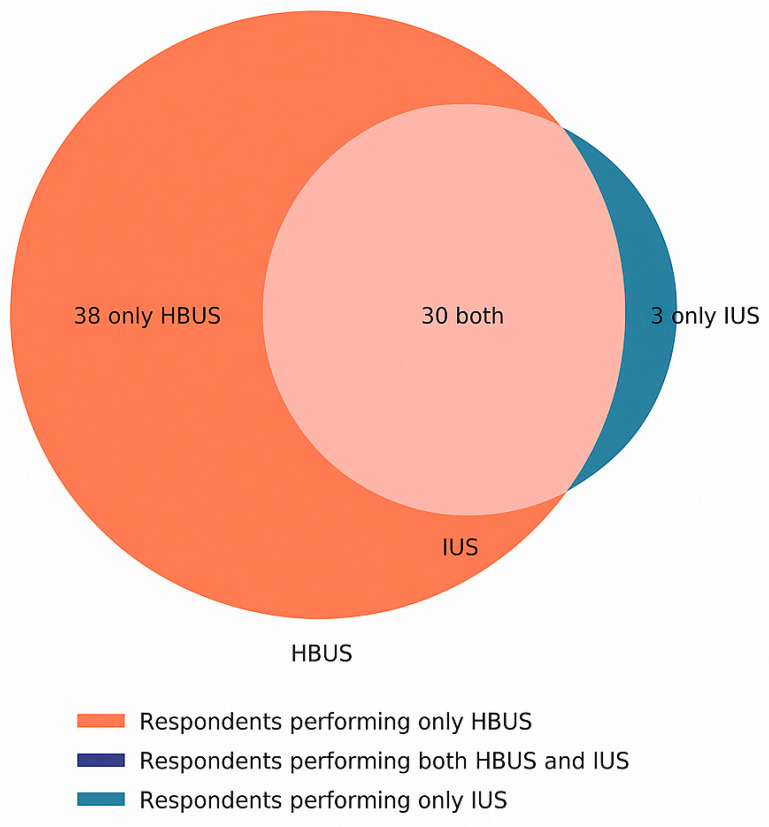
Overlap of respondents performing HBUS and IUS.

A significant overlap was observed between respondents who independently perform HBUS and those performing IUS, with nearly all independent IUS users also engaging in HBUS (Figure 5).

Training in HBUS was reported by 39% (n = 75), with 20% (n = 38) receiving it during their fellowship, 10% (n = 19) through specialized courses, and 9% (n = 18) through both avenues. Of the 75 respondents who reported having received training in HBUS and the 51 who reported training in IUS, 41 individuals indicated receiving training in both modalities. This overlap suggests a significant portion of those trained in one ultrasound technique are also trained in the other.

The primary purposes for using HBUS included monitoring choledocholithiasis, cholangitis or cholecystitis (93%, n = 178), cirrhosis (85%, n = 162), and steatosis (86%, n = 165), reflecting its role in managing hepatic complications in gastroenterology practice.

### 3.4. Comparison of IUS and HBUS Usage Between Pediatric and Adult Gastroenterologists

**Figure 6:**
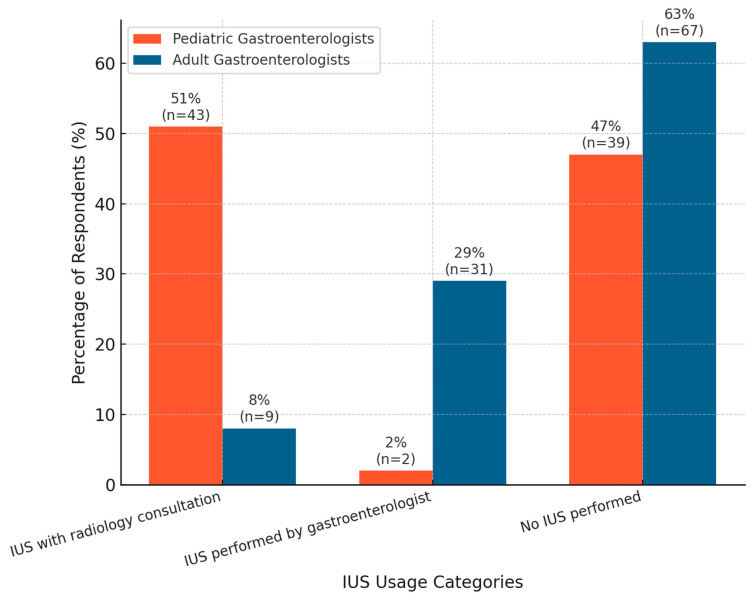
Comparison of IUS usage between pediatric and adult gastroenterologists.

Significant differences were found in IUS usage patterns between pediatric and adult gastroenterologists (*p* < 0.001). Pediatric gastroenterologists were more likely to use IUS with radiology consultation, while adult gastroenterologists more commonly performed IUS independently. A substantial proportion of respondents in both groups reported not using IUS, as shown in Figure 6.

The use of HBUS also varied significantly between the two groups. Pediatric gastroenterologists were more likely to request radiology consultation for HBUS (70%, n = 59) compared to adult gastroenterologists (23%, n = 25), with a statistically significant difference (*p* < 0.001). On the other hand, adult gastroenterologists performed HBUS themselves more frequently (61%, n = 65) compared to pediatric gastroenterologists (4%, n = 3). When compared with IUS, fewer pediatric gastroenterologists (26%, n = 22) and adult gastroenterologists (16%, n = 17) reported not using HBUS.

Among pediatric gastroenterologists, 78% (n = 21) reported not feeling proficient in performing IUS, compared to 34% (n = 23) of adult gastroenterologists, reflecting a statistically significant difference (*p* < 0.001). Conversely, a greater proportion of adult gastroenterologists, 66% (n = 45), reported feeling proficient in performing IUS, compared to only 22% (n = 6) of their pediatric counterparts.

For HBUS, a similar trend was observed. Among pediatric gastroenterologists, 57% (n = 36) indicated that they did not feel proficient, whereas this figure was significantly lower among adult gastroenterologists, at 15% (n = 16). On the other hand, 85% (n = 88) of adult gastroenterologists reported feeling proficient in HBUS, compared to 43% (n = 27) of pediatric gastroenterologists (*p* < 0.001).

The frequency of IUS usage was compared between pediatric and adult gastroenterologists. Among pediatric gastroenterologists, 13% (n = 6) reported using IUS rarely, 49% (n = 22) frequently, and 38% (n = 17) for every patient. In comparison, 30% (n = 12) of adult gastroenterologists used IUS rarely, 45% (n = 18) frequently, and 25% (n = 10) for every patient. Overall, the distribution of usage patterns showed differences between the two groups; however, the association was not statistically significant (*p* = 0.140).

The purposes of using IUS were analyzed and compared between pediatric and adult gastroenterologists, revealing statistically significant differences. Pediatric gastroenterologists reported using IUS for diagnostic purposes more frequently (80%, n = 36) compared to adult gastroenterologists (45%, n = 18), with the difference being statistically significant (*p* < 0.001). Similarly, a significant association was observed in the use of IUS to assess treatment response, with 71% (n = 32) of pediatric gastroenterologists using it for this purpose compared to 90% (n = 36) of adult gastroenterologists (*p* = 0.030). Additionally, IUS was used for identifying complications by 89% (n = 40) of pediatric gastroenterologists, which was significantly higher than the 65% (n = 26) reported by adult gastroenterologists (*p* = 0.008). These results indicate notable differences in the specific purposes for which IUS is utilized between the two groups.

When comparing the challenges faced by pediatric and adult gastroenterologists in using IUS, significant differences were observed in several areas. Lack of training opportunities was a more frequently reported challenge among pediatric gastroenterologists (87%, n = 73) compared to their adult counterparts (67%, n = 72) (*p* = 0.002). Similarly, insufficient equipment was reported by 49% (n = 41) of pediatric gastroenterologists, significantly higher than the 31% (n = 33) reported by adult gastroenterologists (*p* = 0.011). Difficulty interpreting images was also noted more commonly among pediatric gastroenterologists (52%, n = 44) than adult gastroenterologists (34%, n = 36) (*p* = 0.009). In contrast, time constraints were reported by 42% (n = 35) of pediatric and 50% (n = 54) of adult gastroenterologists, with no statistically significant difference between the groups (*p* = 0.226).

## 4. Discussion

IUS has become an indispensable tool for managing IBD, offering unique advantages compared to other imaging modalities such as MRE and CTE. One of IUS’s most notable benefits is its non-invasive nature, which allows for repeated assessments without the need for ionizing radiation. This aspect is especially crucial for IBD patients, many of whom require frequent monitoring due to the chronic and relapsing nature of conditions such as Crohn’s disease and ulcerative colitis [9]. Furthermore, IUS has shown diagnostic accuracy comparable to that of MRE in detecting transmural inflammation, strictures, and abscesses, which are common in CD patients [10]. Beyond its accuracy, IUS provides real-time, point-of-care insights, making it invaluable in clinical settings where rapid decision-making is essential. Unlike MRE, which often requires scheduling and waiting times for both the scan and the results, IUS can be performed at the bedside, allowing clinicians to immediately assess treatment response and adjust management strategies as needed [11]. Additionally, IUS is more cost-effective than MRE or CTE, with studies indicating substantial cost savings when IUS is incorporated into standard IBD care pathways [12]. This financial accessibility further underscores IUS’s suitability for broader implementation, particularly in regions with limited healthcare resources.

From a patient-centered perspective, IUS is highly tolerated and generally well-accepted, as it avoids the discomfort associated with bowel preparation and sedation, which are often required for colonoscopy and MRE. This is particularly advantageous for pediatric populations, where non-invasive, stress-free diagnostic tools are preferred [13]. IUS is increasingly recommended for pediatric patients with IBD due to its non-invasive nature and the ability to reduce radiation exposure, which is especially important for children. Current guidelines emphasize the role of IUS not only in the initial diagnosis but also in the ongoing management of pediatric IBD, as it offers a reliable assessment of disease activity and extent without the risks associated with repeated exposure to radiation from other imaging methods. By minimizing invasive procedures, IUS provides a child-friendly approach that aligns with the goals of safety and comfort in pediatric care [14]. The dynamic nature of IUS, which enables visualization of bowel wall thickness, vascularity, and motility in real-time, also contributes to its value in assessing disease activity and complications. This capability allows IUS to detect changes in bowel wall structure and vascular flow, markers of both acute inflammation and chronic damage, with accuracy comparable to that of cross-sectional imaging [15,16,17]. As IBD management continues to shift towards a treat-to-target approach, where achieving both clinical and endoscopic remission is essential, IUS emerges as a vital tool that aligns well with these goals, supporting frequent, low-risk monitoring.

While IUS has been validated as an effective and patient-centered tool for managing IBD, its adoption varies significantly across regions. In countries such as Germany and Italy, IUS has become an integral part of IBD management, supported by structured training programs that have set standards for its use and accuracy. For instance, in Germany, gastroenterologists are required to perform hundreds of supervised abdominal ultrasounds, including IUS, as part of their training, ensuring high competency levels among specialists. Italy has also incorporated IUS into routine IBD management within outpatient settings, where it is used alongside physical examinations and laboratory results to inform treatment decisions. This integration minimizes the need for patients to visit radiology departments, reducing costs and enhancing convenience [18]. In contrast, countries such as the United Kingdom primarily rely on MRE and CTE, with IUS limited to select centers; ensuring high-quality IUS requires dedicated training in gastroenterology programs [19]. Of note, a North American survey highlighted that most pediatric gastroenterologists were interested in IUS but identified limited training and high inter-observer variability as key barriers [11]. Although the total number of participants represents only a fraction of all gastroenterologists in Turkey, our sample predominantly comprises clinicians working at tertiary-care centers, such as university hospitals and training and research institutions. These centers are the most likely environments for the adoption and promotion of IUS, particularly in managing complex IBD cases. A substantial proportion of respondents also reported a special interest in IBD or hepatology. Since multiple specialties could be selected, this reflects the common overlap in real-world gastroenterology practice.

In our survey on IUS utilization, we included questions on HBUS usage, reflecting our hypothesis that gastroenterologists are generally more familiar with this modality due to greater exposure during their formal training. European gastroenterology curricula emphasize abdominal ultrasound proficiency and ultrasound-guided liver biopsies as foundational skills [20,21], which are particularly relevant in Turkey, where liver biopsies are frequently performed using ultrasound [22,23]. This training and routine application likely contribute to the broader practice and higher proficiency in HBUS compared to IUS. The survey also revealed a disparity in ultrasound proficiency, with adult gastroenterologists reporting higher confidence than their pediatric counterparts, likely due to greater hands-on experience during fellowship. While most respondents had fewer than six years of professional experience, this represents a strategic opportunity. In Turkey, gastroenterology fellowship comprises a structured three-year training program and currently represents the only formal period during which ultrasonography training can be integrated into clinical education. There is no subspecialty training available for advanced IBD beyond fellowship. Consequently, early-career clinicians appear to us to represent the cohort whose awareness, motivation, and access to training should be supported to ensure the long-term, nationwide adoption of IUS. Additionally, the high representation of pediatric gastroenterologists strengthens the study, as pediatric and adult IBD care are increasingly interconnected. Many patients transition from pediatric to adult care around the age of 18, and shared follow-up during this transition is common. We believe that pediatric and adult IBD care are interconnected rather than operating separately.

Based on the survey results, well-established methods such as colonoscopy, fecal calprotectin, MRE, and clinical scoring systems are predominantly used for monitoring IBD activity. Although a subset of respondents reported using IUS for every patient, this should not be interpreted as evidence of widespread adoption. Our cohort largely consisted of clinicians based in tertiary centers where IUS is more likely to be available, and even within this group, the majority reported infrequent or no use. Furthermore, the observation that many frequent users did not feel proficient underscores the gap between clinical demand and formal training, reinforcing the urgent need for structured educational programs.

The ECCO e-Quality project provides a comprehensive framework to ensure consistent and high-quality care for IBD patients across Europe. One of its key recommendations highlights the need for at least two imaging techniques [MR or CT enterography or bowel ultrasound] to assess disease activity and complications [24]. Our study evaluates the current utilization of IUS in Turkey, revealing that, despite its inclusion in international guidelines as a recommended imaging modality, IUS remains underutilized due to factors such as limited training opportunities, lack of national guidelines, and varying levels of proficiency among gastroenterologists. These barriers underscore the importance of structured educational initiatives and standardized protocols to better integrate IUS into clinical practice and align Turkey’s practices with ECCO recommendations.

The International Bowel Ultrasound Group (IBUS) has established a comprehensive and internationally recognized curriculum in gastrointestinal ultrasound (GIUS) training, structured across three modules. Module 1 introduces GIUS through an intensive didactic course and hands-on workshop, while Module 2 provides four weeks of clinical training at IBUS-certified centers, allowing trainees to document over 200 GIUS cases. Module 3 concludes with an advanced workshop and final assessment at major conferences such as ECCO and Digestive Disease Week (DDW) [25]. Establishing a similar training structure in Turkey could significantly enhance local GIUS proficiency, particularly in IBD management.

Encouraging Turkish gastroenterologists to participate in IBUS programs, supported by financial aid and educational agreements with international centers, could further strengthen IUS training. Selected fellows could benefit from 3- to 6-month opportunities abroad, facilitated by the Turkish Society of Gastroenterology. To measure the impact of these initiatives, a follow-up survey in 3 to 5 years could benchmark progress in IUS utilization and proficiency, using the current survey as a baseline.

## 5. Conclusions

IUS offers a patient-centered, cost-effective imaging option for IBD management, reducing reliance on invasive procedures and radiation exposure while providing real-time insights into disease activity. Despite its widespread use in some European countries, limited training and the lack of standardized guidelines hinder broader adoption. Our survey, the first in Turkey, highlighted these barriers and underscored the need for structured educational programs and national guidelines. While the study’s limited reach may have constrained its findings, it provides valuable insights to guide future training initiatives and facilitate the integration of IUS into routine clinical practice, both in Turkey and globally.

## Figures and Tables

**Table 1:** Demographic characteristics of participants.

Characteristic	n (%)
Total Gastroenterologists	191
Adult Gastroenterologists	107 (56%)
Pediatric Gastroenterologists	84 (44%)
Age (years)	
Under 40	46 (24.1%)
40–50	87 (45.5%)
50–60	41 (21.5%)
Over 60	17 (8.9%)
Gender	
Male	104 (54.5%)
Female	87 (45.5%)
Professional Experience (years)	
Less than 3 years	59 (30.9%)
3–6 years	43 (22.5%)
6–10 years	15 (7.9%)
Over 10 years	74 (38.7%)
Academic Titles	
Fellow	127 (63.1%)
Specialist	30 (15.7%)
Associate Professor	34 (17.8%)
Professor	6 (3.4%)
Special Interest	
Inflammatory Bowel Disease	94 (49.2%)
Hepatology	78 (40.8%)
Institution Type	
University Hospital	87 (45.5%)
Training and Research Hospital	65 (34.0%)
Private Clinic	29 (15.2%)
Private Practice	11 (5.8%)
State Hospital	14 (7.3%)

## Data Availability

The data presented in this study are available on request from the corresponding author. The data are not publicly available due to privacy and ethical restrictions.

## Supplementary Materials

The following supporting information can be downloaded at: https://www.mdpi.com/article/10.3390/jcm14144817/s1, Figure S1: Survey Process Flow for Gastroenterologists in Turkey (Nov 1–Nov 14, 2024); Table S1: Survey Questions.
