# Supplementary material for: Intestinal Ultrasound: Advancing Towards Broader Adoption—Insights from a National Survey in Turkey
Source: J Clin Med. 2025 Jul 8;14(14):4817. doi: 10.3390/jcm14144817 (PMC12296133; doi:10.3390/jcm14144817)
Supplement: Supplementary file 1 [file jcm-14-04817-s001.zip › jcm-3678882-supplementary.pdf]

Figure S1:

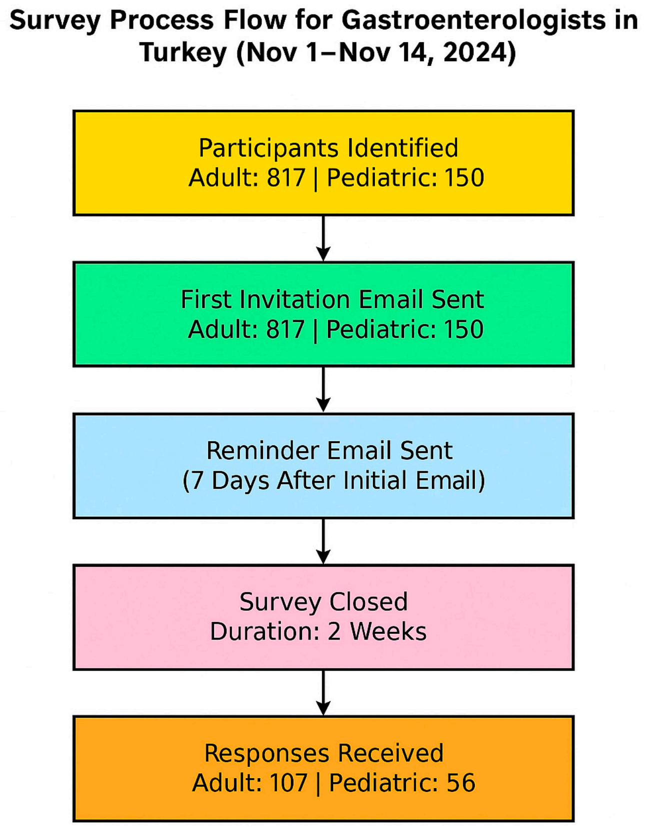

Table S1: Survey Questions

Question Number	Question Text
1	What is your age? (<40, 40-50, 50-60, >60)
2	What is your gender? (Male, Female)
3	What is your medical specialty? (Adult Gastroenterologist, Pediatric Gastroenterologist)
4	How many years of experience do you have in gastroenterology? (>3, 3-6, 6-10, >10)
5	What is your special area of interest? (Inflammatory Bowel Disease, Hepatology, Advanced Endoscopy, None)
7	What is your academic title? (Fellow, Specialist, Associate Professor, Professor)
8	Approximately how many IBD patients do you examine per month? (<10, 10-30, >30)
9	Which imaging methods, in addition to endoscopy, do you use for diagnosing IBD? (MRE, CT, Intestinal Ultrasound, Combination of Intestinal Ultrasound with MRE or CT)

10	What tests, imaging methods, or scoring systems do you use for monitoring IBD treatment? (Colonoscopy, Fecal Calprotectin, MRE, Intestinal Ultrasound, Clinical Scoring such as Ulcerative Colitis activity index and Crohn's disease activity index))
11	Do you use Intestinal Ultrasound? (Yes, I perform it myself; Yes, I request a radiology consultation; No, I do not use it)
12	How frequently do you use Intestinal Ultrasound? (Rarely, Frequently, For every patient)
13	For what purpose do you use Intestinal Ultrasound? (Diagnosis, Treatment Response, Identification of Complications)
14	Have you received training in Intestinal Ultrasound? (Yes, No)
15	Where did you receive your training in Intestinal Ultrasound? (During fellowship, Through a course, Both)
16	How proficient do you feel in using Intestinal Ultrasound? (Not proficient, Somewhat proficient, Moderately proficient, Fully proficient, Very proficient)
17	What do you consider the main advantage(s) of Intestinal Ultrasound in diagnosing and monitoring treatment response in IBD? (Non-invasive, Cost-effective, Real-time Imaging, Lack of radiation exposure, Ability to visualize bowel wall layers and vascularity)
18	What is the main challenge you face when using Intestinal Ultrasound? (Difficulty in interpreting ultrasound findings, Lack of training, Insufficient equipment, Time constraints)
19	Do you use Hepatobiliary Ultrasound? (Yes, No)
20	How frequently do you use Hepatobiliary Ultrasound? (Rarely, Frequently, For every patient)
21	Have you received training in Hepatobiliary Ultrasound? (Yes, No)
22	Where did you receive your training in Hepatobiliary Ultrasound? (During fellowship, Through a course, Both)
23	In which clinical situations do you use Hepatobiliary Ultrasound? (Monitoring choledocholithiasis, Cholangitis or Cholecystitis, Cirrhosis, Steatosis, and others)
24	How proficient do you feel in using Hepatobiliary Ultrasound? (Not proficient, Somewhat proficient, Moderately proficient, Very proficient)
25	What do you think should be done to promote the widespread adoption of Intestinal Ultrasound in Turkey? (Increasing educational opportunities, Financial support for equipment, Organizing courses, Establishing IUS units in hospitals)
26	If an Intestinal Ultrasound course were organized in Turkey, would you be interested in attending? (Yes, No)
